# 30-Year Experience With 22 Cases of Malignant Transformation Arising From Ovarian Mature Cystic Teratoma: A Rare Disease

**DOI:** 10.3389/fonc.2022.842703

**Published:** 2022-05-09

**Authors:** Yan Li, Meng Qin, Ying Shan, Huan-wen Wu, Xiao-ding Liu, Jie Yin, Yu Gu, Wei Wang, Yong-xue Wang, Jia-yu Chen, Li Ma, Ying Jin, Ling-ya Pan

**Affiliations:** ^1^ Department of Obstetrics and Gynecology, Peking Union Medical College Hospital, Chinese Academy of Medical Sciences and Peking Union Medical College, National Clinical Research Center for Obstetric & Gynecologic Diseases, Beijing, China; ^2^ Department of Pathology, Peking Union Medical College Hospital, Chinese Academy of Medical Sciences and Peking Union Medical College, Beijing, China; ^3^ Department of Anesthesiology, Peking Union Medical College Hospital, Chinese Academy of Medical Sciences and Peking Union Medical College, Beijing, China

**Keywords:** ovarian mature cystic teratoma, malignant transformation, survival outcomes, squamous cell carcinoma, rare disease (RD)

## Abstract

**Objective:**

To investigate the clinical characteristics and survival outcomes of patients with malignant transformation arising from ovarian mature cystic teratoma (MT-MCT).

**Methods:**

This retrospective study included patients with ovarian MCTs at Peking Union Medical College Hospital (PUMCH) during 1990.01-2020.12. When the pathologic histology was MT-MCT, detailed information was collected.

**Results:**

Overall, 7229 ovarian MCT patients and 22 patients with MT-MCT were enrolled. The rate of malignant transformation of all ovarian MCTs was 0.30%. Most patients with MT-MCT were 51 (21–75) years old, and the tumor mass size was 10 (3–30) cm. The typical clinical symptoms were mainly abdominal pain and distension. The levels of tumor markers were elevated on preoperative examination. Early diagnosis could be made by ultrasonic examination, pelvic enhanced MRI and CT. Most patients underwent debulking surgery and adjuvant chemotherapy. The most common histological type to exhibit malignant transformation was squamous cell carcinoma (59.1%), followed by adenocarcinoma (13.6%), carcinoid (9.1%), and borderline tumor (18.2%). The 5-year RFS and OS rates were 54.5% and 81.8%, respectively. Patients with FIGO stage I had the best RFS (P=0.047) and OS (P=0.018), followed by those with FIGO stage II-IV.

**Conclusion:**

MT-MCTs mainly occur in elderly females, are rare and have a poor prognosis. Advanced FIGO stage is a risk factor for survival. Although there is no standard treatment, cytoreductive debulking surgery and adjuvant chemotherapy could be considered. Perimenopausal and menopausal women with MCT should receive surgical treatment.

## Introduction

Ovarian germ cell tumors (OGCTs) are derived from ovarian primordial germ cells, including benign or malignant tumors. OGCTs account for approximately 20%-25% of all ovarian tumors but only approximately 5% of all ovarian malignant tumors (1, 2). OGCTs mainly occur in young women aged 10–30 years, accounting for 70% of female ovarian tumors in this age group (3). Mature cystic teratomas (MCTs), also referred to as dermoid cysts, are one of the most common benign OGCTs (4). The potential cells in teratomas can differentiate into a variety of tissues from the three layers of primordial germ cells.

When the components of MCTs develop into somatic malignant tumors, they can become malignant OGCTs, which is called malignant transformation arising from ovarian mature cystic teratomas (MT-MCTs) (5, 6). It is reported that the incidence of malignant transformation of MCTs is 0.2%-2% (6, 7), accounting for 2.9% of all malignant OGCTs (8). Any component of MCTs can progress to malignancy, but the most common malignant transformation type is squamous cell carcinoma (SCC) from the ectoderm (9, 10). MT-MCTs are not equal to ovarian immature teratomas (IT) (11). Immature teratomas are also known as malignant teratomas, teratoblastomas or embryonal teratomas (12) and account for less than 1% of ovarian teratomas but 35.6% of all malignant OGCTs. IT patients are often under 20 years of age (13), while MT-MCTs were more common in women of postmenopausal age (14).

The preoperative detection of MT-MCT is very challenging. The chief symptoms and positive tumor markers lack specificity and sensitivity (5). The early diagnosis of teratomas mainly relies on ultrasonic examination (15). The echo of MCT on ultrasound is obviously related to its structure. In addition, magnetic resonance imaging (MRI) and computed tomography (CT) scans have better efficacy and specificity in tumor location and qualitative diagnosis than ultrasound (16, 17). However, it is still difficult to distinguish MT-MCT from other benign and malignant ovarian tumors. A few studies ever reported that risk factors included age ≥45 years, tumor size >10 cm, rapid growth, and imaging findings (10, 18, 19). There are no standard treatment strategies about MT-MCT. Typical treatment is complete debulking surgery followed by adjuvant chemotherapy (19). When malignant transformation has occurred in the teratoma, the treatments always adjust according to the type of transformation (19).

However, as MT-MCT is a rare disease, there are almost no evidence-based studies. Therefore, we performed this retrospective study to explore the characteristics and clinical outcomes of patients with malignant transformation arising from ovarian mature cystic teratoma.

## Materials and Methods

### Study Population

This retrospective study included all patients with ovarian MCTs between January 1990 and December 2020 at Peking Union Medical College Hospital (PUMCH). Patients with any of the following characteristics were excluded: 1) critical clinical or operation data were lacking; or 2) repeated specimen collection was required. All patients provided written informed consent under the approval of the Ethics Committee of PUMCH. All ethical standards, including ethics committee approval and the informed consent procedure, were in accordance with international guidelines.

### Data Collection

The basic information and pathologic histology of ovarian MCT patients were extracted from the medical records from the Hospital Information System (HIS). When the pathologic histology revealed MT-MCT, detailed information was collected. For this retrospective analysis, the following data were extracted from HIS and through telephone interviews: patient information, clinical information, pathological characteristics, surgical outcomes, and survival outcomes. Two pathologists who have abundant experience in gynecologic pathology independently examined the slides with hematoxylin-eosin (HE) staining and immunohistochemistry for all included patients. A third reviewer was involved in a discussion to resolve differences. The standard tumor stage was defined by the International Federation of Gynecology and Obstetrics (FIGO) staging system in 2014. The recurrence-free survival (RFS) was defined as the time interval between the date of the first diagnosis and the date of ovarian cancer progression. The overall survival (OS) was defined as the time interval between the date of the first diagnosis and the date of death (20). RFS and OS were the survival prognosis indices for this study.

### Statistical Analysis

All statistical analyses were performed using SPSS software (version 23.0; SPSS Inc., Chicago, IL, USA), and graphs were generated using GraphPad Prism software for Macbook (version 9.0; GraphPad software Inc., San Diego, USA). Student’s t tests and Mann–Whitney U tests were used to compare continuous variables. Pearson’s chi-squared tests and Fisher’s exact tests were used to compare categorical variables (21). Survival analysis was performed using Kaplan–Meier curves and the log-rank test. Statistical significance was set at *P*<0.050.

## Results

Overall, 7229 ovarian MCT patients were diagnosed at PUMCH during the period from 1990.01-2020.12. According to the pathological histology results, 22 patients had malignant transformation arising from ovarian MCTs. The rate of malignant transformation in all ovarian MCTs was 0.30%. **Table 1** shows the clinicopathological characteristics and survival outcomes of each patient with MT-MCTs. **Table 2** shows a summary of the clinicopathological characteristics of all patients with MT-MCTs.

**Table 1:** The clinicopathological characteristics and survival outcomes of each patient with malignant transformation arising from ovarian mature cystic teratomas.

Case	Age (yr)	Gestation/pregnancy	Complications	Symptoms	Tumor size (cm)	Positive tumor marker	Neoadjuvant chemotherapy	FIGOstage	Primary Surgery	Malignant transformation Histology	Tumor grade	Adjuvant treatment	Status	Follow- up time(Mo)
1	60	G7P7	None	Pelvic mass	5.0	None	No	III	Cytoreduction	AC-MCT	G1	Yes	Dead	120.6
2	35	G3P1	None	Abdominal distension	3.0	None	No	II	Cytoreduction	BD-MCT	NR	No	Alive	315.4
3	46	G2P2	None	Bellyache	12.0	None	No	IA	Cytoreduction	SCC-MCT	G2	No	Alive	197.7
4	65	G2P1	Nephritis	Bellyache	11.3	None	No	III	Cytoreduction	SCC-MCT	G1	No	Dead	145.4
5	55	G0P0	HTN	Pelvic mass	8.5	Ca125/CA199	Yes	III	Cytoreduction	Goiter-carcinoid	G1	Yes	Dead	41.2
6	71	G1P1	DM	Poor appetite	10.0	Ca125/CA199	No	IIB	Cytoreduction	SCC-MCT	G1	No	Dead	75.5
7	75	G2P2	HTN	Abdominal distension	15.0	CA125	Yes	IIB	Cytoreduction	AC-MCT	G3	Yes	Dead	85.6
8	64	G4P3	None	Pelvic mass	13.3	CA199	No	I	Cytoreduction	BD-MCT	NR	No	Alive	14.1
9	57	G3P1	None	Bellyache	10.0	Ca125/CA199	No	IIB	Cytoreduction	SCC-MCT	G3	Yes	Alive	133.2
10	45	G3P3	DM	Pelvic mass	8.1	Ca125/SCCAg	No	IIIC	Cytoreduction	SCC-MCT	G3	Yes	Dead	16.8
11	41	G1P1	None	Pelvic mass	5.7	Ca125/CA199/CEA	No	II	Oophorocystectomy	BD-MCT	NR	Yes	Recurrent but alive	119.6
12	60	G3P1	None	Vaginocele	12.0	None	No	II	Cytoreduction	SCC	G2	Yes	Dead	114.0
13	33	G2P0	HBV	Abdominal distension	30.0	CA125/CEA/AFP	No	I	Staging Surgery	BD-MCT	NR	No	Alive	107.8
14	39	G5P1	None	Bellyache	8.2	CA199/CEA	No	IIC	Staging Surgery	SCC-MCT	G1	Yes	Dead	24.0
15	25	G0P0	Psoriasis	Bellyache	15.0	CA199	No	IA	Fertility preservation surgery	SCC-MCT	G1	Yes	Alive	12.5
16	21	G0P0	None	Menstrual disorder	7.0	None	No	IC	Fertility preservation surgery	CC-MCT	NR	No	Alive	59.9
17	64	G2P2	HTN	Bellyache	10.0	CA125/CA199	No	II	Cytoreduction	SCC-MCT	G2	Yes	Alive	48.7
18	48	G2P1	Cerebral infarction	Bellyache	12.8	CA125/CA199/SCCAg/CEA	Yes	IV	Cytoreduction	SCC-MCT	G3	Yes	Dead	23
19	62	G3P1	Carotid plaque	Bellyache	8.7	CA125/CA199/SCCAg	No	IA	Staging Surgery	SCC-MCT	G2	Yes	Recurrent but alive	39.9
20	36	G0P0	IgA nephropathy	Bellyache	5.0	CA125/SCCAg	No	IA	Staging Surgery	SCC-MCT	G2	Yes	Alive	11.8
21	62	G1P1	None	Pelvic mass	8.4	Ca125/CA199	No	II	Abdominal hysterectomy+bilateral salpingo-oophorectomy	SCC-MCT	G2	Yes	Alive	7.5
22	46	G2P1	None	Vaginal drainage	14.0	Ca125/CA199/CEA	No	III	Cytoreduction	AC-MCT	G1	Yes	Alive	1.1

**Table 2:** The clinicopathological characteristics summary of all patients with malignant transformation arising from ovarian mature cystic teratomas.

Variable	Number (N = 22)	%	Variable	Number (N = 22)	%
**Age (year)**	51 (21-75)		**Primary Surgery**		
<60	13	59.10%	Cytoreductive surgery	14	63.60%
≥60	9	40.90%	Staging Surgery	4	18.20%
**Complications**			Fertility preservation surgery	2	9.10%
No	11	50.00%	Others	2	9.10%
Yes	11	50.00%	**Reexplore laparotomy for complete stage**		
**Menopause**			No	18	81.80%
No	10	45.50%	Yes	4	18.2
Yes	12	54.50%	**Operation complication**		
**Previous pregnancy**			No	19	86.40%
No	4	18.20%	Yes	3	13.60%
Yes	18	81.80%	intestinal obstruction	1	4.50%
**Previous delivery**			infection	1	4.50%
No	5	22.70%	lymphedema	1	4.50%
Yes	17	77.30%	**Adjuvant treatment**		
**Clinical symptoms**			No	7	31.80%
Bellyache/abdominal distension	12	54.50%	Yes	15	68.20%
Pelvic mass	6	27.30%	TC	8	36.40%
Others	4	18.20%	TP	5	22.70%
**Tumor mass size (cm)**	10 (3-30)		PEB	1	4.50%
<10cm	10	45.50%	Others	1	4.50%
≥10cm	12	54.50%	**Malignant transformation Histology**		
**Positive tumor marker**			Squamous-cell carcinoma	13	59.10%
CA125	13	59.10%	Adenocarcinoma	3	13.60%
CA199	12	54.50%	Carcinoid	2	9.10%
SCCAg	4	18.20%	Borderline tumor	4	18.20%
CEA	5	22.70%	**Tumor grade**		
AFP	1	4.50%	G1	7	31.80%
**Neoadjuvant chemotherapy**			G2	6	27.30%
No	19	86.40%	G3	4	18.20%
Yes	3	13.60%	No report	5	22.70%
**FIGO stage**			**Recurrence**		
I	7	31.80%	No	12	54.50%
II	9	40.90%	Yes	11	50.00%
III	5	22.70%	Recurrent death	9	40.90%
IV	1	4.50%	Recurrent but alive	2	9.10%
			**Follow-up time (Months)**	54.3(12.0-315.4)	

Data are presented as number (%) or mean (±SD) or median (±IQR).

### Clinical Characteristics of the Included Patients

The age of the included patients was 51 (21–75) years old. Half of the patients experience complications, mainly hypertension (HTN), diabetes mellitus (DM), nephritis, etc. The chief complaint was abdominal pain and distension for approximately 1–3 months in 54.5% of patients; 27.3% patients visited a doctor due to a palpable abdominal mass. The tumor mass size of the MCT with malignant transformation was 10 (3–30) cm, which well explained the main symptoms. The tumor mass size of 54.5% of the patients was larger than 10 cm. Other symptoms included irregular menstruation and vaginal discharge. The level of tumor markers was elevated on preoperative examination and mainly included carbohydrate antigen 125 (CA125), carbohydrate antigen 199 (CA199), and squamous cell carcinoma antigen (SCCAg).

**Figure 1:**
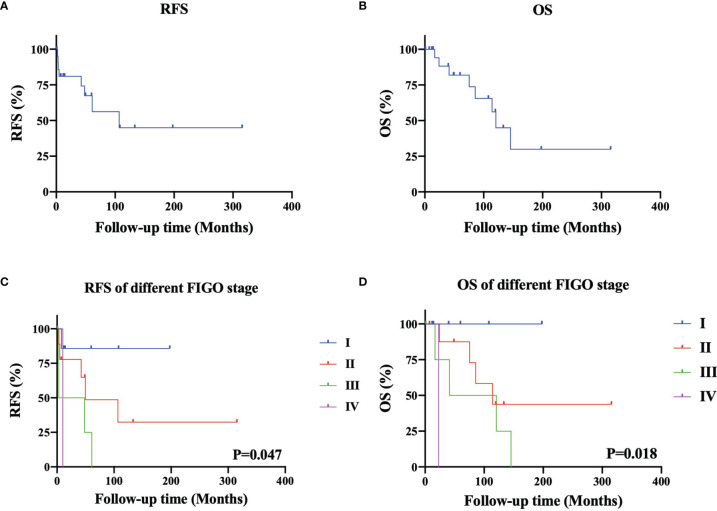
Typical imaging and pathological image of patients with squamous cell carcinoma arising from ovarian mature cystic teretomas (SCC-MCTs). **(A)** [case 18]: Pelvic ultrasound of SCC-MCT showing that a moderate echo was observed in the right adnexal area (10.5*7.9*8.3 cm), with an irregular shape and clear boundry. No echo or strip-shaped strong echo was observed in this area. CDFI: Striped asteriovenous blood flow is seen in the surrounding interior PSV: 11.8 cm/s, RI: 0.35. **(B)** [case 20]: Pelvic enchanced MRI of SCC-MCTs showing a lobulated cystic solid mass from the adnexal area with a high-low mixed signal located in the lower abdomen and pelvic cavity (13.8*8.8*105cm) and compression changes in the adjacent bowel, bladder and uterus. **(C)** [case 18]: Pelvic enchance CT of SCC-MCTs showing a lobulated soft tissue density mass in the pelvic cavity (9.4*9.9cm), with uneven internal density, spot-shaped high-density foci and fat density shadows. In addition, the plain CT value was approximately 37HU. Uneven mild enhancement was observed on enchanced scan. The boundry was not clear between this mass and the small intestine and interior uterine edge in the pelvic cavity. **(D)** [case 18]: Pathological images showing that the gray nodule (9*7*5cm) on the section was cystic and solid. Hairs can be seen in the cystic area, while the solid area appears gray-yellow and solid with medium texture. The tumor consisted of 30-40% malignant components.)

The typical imaging of patients with squamous cell carcinoma transformation in MCTs (SCC-MCTs) is shown in **Figure 1**. The ultrasound and enhanced CT images of case 18 were obtained. In addition, the pelvic enhanced MRI images of case 20 were collected. **Figure 1A** shows a pelvic ultrasound of SCC-MCT, revealing a moderate echo in the right adnexal area (10.5*7.9*8.3 cm) with an irregular shape and clear boundary. No echo or strip-shaped strong echo was observed in this area. CDFI: Striped arteriovenous blood flow is seen in the surrounding interior (PSV: 11.8 cm/s and RI: 0.35). In **Figure 1B**, the pelvic enhanced MRI of SCC-MCTs showed a lobulated cystic solid mass from the adnexal area with a high-low mixed signal located in the lower abdomen and pelvic cavity (13.8*8.8*10.5 cm) and compression changes in the adjacent bowel, bladder and uterus. In **Figure 1C**, enhanced CT of SCC-MCTs in the pelvis revealed a lobulated soft tissue density mass in the pelvic cavity (9.4*9.9 cm) with uneven internal density, spot-shaped high-density foci and fat density shadow. In addition, the plain CT value was approximately 37 HU. Uneven mild enhancement was observed on the enhanced scan. The boundary was not clear between this mass and the small intestine and anterior uterine edge in the pelvic cavity.

There were four histological types of MT-MCT exhibiting malignant transformation, namely, squamous cell carcinoma (SCC-MCT, 13/22, 59.1%), adenocarcinoma (AC-MCT, 3/22, 13.6%), carcinoid (CC-MCT, 2/22, 9.1%), and borderline tumor (BD-MCT, 4/22, 18.2%). CA125 and SCCAg were usually elevated in SCC-MCTs. The tumor differentiation grade included well differentiated (G1, 31.8%), moderately differentiated (G2, 27.3%) and poorly differentiated (G3, 18.2%). The tumor grade was not reported in CC-MCT and BD-MCT because of its pathological particularity. **Figure 1D** shows a typical pathological image of a patient (case 18) with SCC-MCT. In this case, the gray nodule (9*7*5 cm) appears cystic and solid. Hairs can be seen in the cystic area, while the solid area is gray–yellow and has medium texture. For this case, the tumor consisted of 30-40% malignant components. Most ovarian patients with MT-MCTs were in the early FIGO stage. The number of patients with FIGO stage I-IV disease was seven, nine, five, and one, respectively. One FIGO stage IV patient had bone and lung metastasis.

### Treatments of the Included Patients

Three patients underwent neoadjuvant chemotherapy (NACT). Among them, two patients had extensive implant metastasis in the upper abdomen and a large tumor burden throughout the whole body in preoperative evaluation, thus receiving NACT to achieve a higher rate of satisfactory cytoreduction. The other one patient was at advanced age and had a poor performance status that could not tolerate primary cytoreduction. After receiving NACT, the tumor burden of all three patients were significantly reduced by CA125 and CT scans (22, 23). Among all 22 patients, 90.9% of patients underwent standard ovarian surgery, including cytoreductive surgery (63.6%), ovarian cancer staging surgery (18.2%), and fertility preservation staging surgery (9.1%). Only one patient underwent total abdominal hysterectomy and bilateral salpingo-oophorectomy, and the other one patient underwent oophorocystectomy. If it’s highly suspected that the pelvic mass was malignant by preoperative evaluation, the immediate rapid pathological examination during operation was necessary. The rate of reexplore laparotomy for complete stage was 18.2%. A total of 13.6% of patients had operation complications, including intestinal obstruction (4.5%), infection (4.5%), and lymphedema (4.5%). A total of 68.2% of patients received adjuvant chemotherapy, including TC (taxol+carboplatin, 8/15), TP (taxol+cisplatin, 5/15), and PEB (cisplatin+ etoposide + bleomycin, 1/15).

### Survival Outcomes of the Included Patients

**Figure 2:**
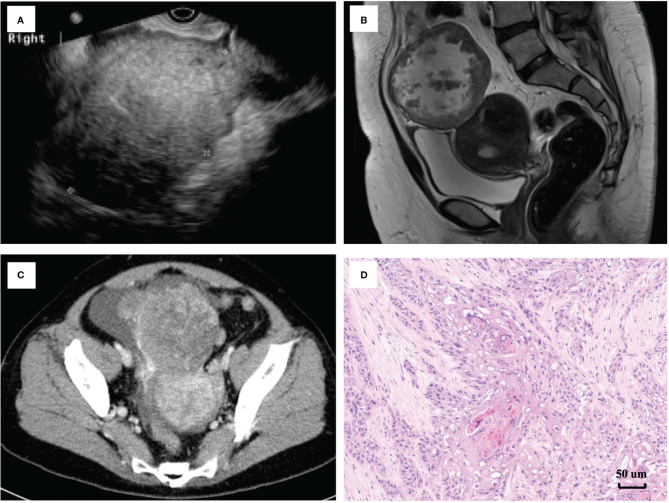
The survival outcomes of patients with malignant transformation arising from ovarian mature cystic teratomas. The recurrence-free survival **(A)** and overall survival **(B)** of 22 patients with MT-MCT. The recurrence-free survival **(C)** and overall survival **(D)** of 22 patients characterized by different FIGO stage).

The follow-up time was 54.3 (12.0-315.4) months. **Figures 2A**, **2B** show the RFS and OS of 22 patients with MT-MCT, respectively. The 3-year and 5-year RFS rates were 72.7% and 54.5%, respectively. The 3-year and 5-year OS rates were 86.4% and 81.8%, respectively. Among all nine dead patients, eight patients were dead due to tumor recurrence, and one patient died of suicide. The recurrence sites were commonly in pelvic cavity and inguinal lymph nodes. In addition, we performed a subgroup analysis of the RFS and OS of 22 patients characterized by FIGO stage, as shown in **Figures 2C, D**. The results showed that patients with FIGO stage I disease had the best RFS, followed by those with FIGO stages II, III, and IV (P=0.047). Similarly, the patients with FIGO stage I disease had the best OS, followed by those with FIGO stage II, III, and IV (P=0.018). The 5-year RFS rates in patients with FIGO stages I, II, III, and IV were 85.7%, 55.6%, 20.0%, and 0%, respectively. The 5-year OS rates in patients with FIGO stages I, II, III, and IV were 100.0%, 88.9%, 60%, and 0%, respectively.

**Table 3** showed the clinicopathological characteristics and survival outcomes of all included patients characterized by SCC-MT group and other histology group. There were no significant differences in all variables, including age, clinical symptoms, tumor mass size, the number of NACT and adjuvant treatment, FIGO stage, primary surgery type, and the number of recurrent patients. We performed a subgroup analysis of the RFS and OS outcomes of 22 patients characterized by different MT histology, as respectively shown in **Figures S1A, B**. The patients with BD-MCT tended to have better survival outcomes than those with SCC-MCT and AC-MCT/CC-MCT. However, there were no significant differences in RFS (P=0.220) and OS (P=0.163). The 5-year RFS rates were respectively 61.5% and 100% between SCC-MCT group and other histology group. The 5-year OS rates were respectively 76.9% and 77.7% between two groups.

**Table 3:** The clinicopathological characteristics and survival outcomes of all included patients characterized by different malignant transformation histology.

Variable	SCC-MT (N = 13)	Other Histology (N = 9)	*P* value
**Age (year)**			0.548
<60	7 (53.8%)	6 (66.7%)	
≥60	6 (46.2%)	3 (33.3%)	
**Clinical symptoms**			0.219
Bellyache/abdominal distension	9 (69.2%)	3 (33.3%)	
Pelvic mass	2 (15.4%)	4 (44.4%)	
Others	2 (15.4%)	2 (22.2%)	
**Tumor mass size (cm)**			0.429
<10cm	5 (38.5%)	5 (55.6%)	
≥10cm	8 (61.5%)	4 (44.4%)	
**Neoadjuvant chemotherapy**			0.329
No	12 (92.3%)	7 (77.8%)	
Yes	1 (7.7%)	2 (22.2%)	
**FIGO stage**			0.643
I	4 (30.8%)	3 (33.3%)	
II	6 (46.2%)	3 (33.3%)	
III	2 (15.4%)	3 (33.3%)	
IV	1 (7.7%)	0	
**Primary Surgery**			0.902
CRS	8 (61.5%)	6 (66.7%)	
Staging Surgery	3 (23.1%)	1 (11.1%)	
Fertility preservation surgery	1 (7.7%)	1 (11.1%)	
TAH+BSO	1 (7.7%)	1 (11.1%)	
**Adjuvant treatment**			0.29
No	3 (23.1%)	4 (44.4%)	
Yes	10 (76.9%)	5 (55.6%)	
**Recurrence**			0.665
No	6 (46.2%)	5 (55.6%)	
Yes	7 (53.8%)	4 (44.4%)	
Recurrent death	6 (85.7%)	3 (75.0%)	
Recurrent but alive	1 (14.3%)	1 (25.0%)	
**5-year RFS rates**	61.50%	100%	
**5-year OS rates**	76.90%	77.70%	

## Discussion

MT-MCT is a rare type of malignant ovarian cancer (24). Ovarian MCTs mainly occur in women during the child-bearing years, while types that exhibit malignant transformation generally occur in women between 40 and 60 years old (25, 26). It has been reported that the average age of patients with MT-MCTs is 50 years old, while the average age of patients with benign teratomas is 33 years old (9). MT-MCT tumors are typically located in one ovary but can involve both ovaries. The tumor mass size is usually large, most of which are 10 to 20 cm in diameter (27). The level of positive tumor markers is associated with MT-MCTs, and different tumor markers have different sensitivities for different histological types (28). Thus, it is difficult to identify these tumors using a single tumor marker. In this study, the clinical characteristics of patients with MT-MCTs were consistent with the findings from other literature reports, including age, symptoms, tumor mass size, and positive tumor markers.

Early diagnosis of MT-MCT relies on comprehensive imaging examinations including ultrasound, MRI, and CT (29). A large number of lipids, hair, cartilage and thyroid cells cause MCTs to exhibit obvious ultrasonic imaging characteristics, such as the dough sign, wall nipple sign and lipid stratification sign (18). However, lipid stratification and calcification are also present in MT-MCTs. MRI showed that the thickening of the cyst wall and the presence of intracapsular papilla and solid components can be important features in the diagnosis of malignant MCTs (16). In addition, direct invasion of the surrounding tissues or peritoneal implantation metastasis is observed in some cases. The distinguishing features of MT-MCT are the presence of components of benign teratoma (e.g., grease, hair, bone, etc.) and a combination of malignant tumor features (e.g., blood flow signals or enhanced solid components). Therefore, it is important to identify older patients with larger tumors or those who have tumors with malignant characteristics to exclude potential malignant transformation of MCTs (27). It is emphasized that surgical treatment should be performed for perimenopausal and postmenopausal patients with ovarian mature teratoma, even though almost all of them are benign.

The pathological diagnosis of MT-MCTs is difficult. Pathologists should have abundant experience in diagnosing gynecologic tumor pathologies. Finding a teratomatous component is critical. Malignant transformation may occur in the endodermal, mesodermal and exodermal components of teratomas (30). In terms of pathological characteristics, ovarian MCTs are generally large and solid, with or without a dermoid cyst. They may protrude into the cyst wall or form thickening of the wall (31). It is a grayish white, raised or nodular, lumpy, papillary and cauliflower mass. It is often brittle, accompanied by bleeding and necrosis (32). SCC is the most common malignant transformation histology of MCTs (33). SCC-MCT shows a varied morphology, ranging from G1 and keratinizing to G3 to anaplastic (31). One study reported that squamous carcinoma of MCT originates from squamous epithelial metaplasia, which may be related to HPV infection (30). AC-MCT most commonly arise from gastrointestinal-type epithelium and respiratory-type epithelium. Low-grade mucinous epithelial neoplasms with mucin extravasation may arise in teratomas and mimic metastases (31). The tumor consisted of 10-40% malignant components, which depends on the histology type and tumor grade. Other rare types include thyroid-type papillary carcinoma, undifferentiated carcinoma, borderline tumor, melanoma, small cell carcinoma, and sarcomas (34–39). The immunophenotype of the malignant components is similar to that of malignancies occurring at classical sites. In addition, it is necessary to identify the difference between ovarian metastatic tumors and MT-MCTs. Doctors should exclude metastasis of cervical or vaginal squamous cell carcinoma, gastrointestinal adenocarcinoma, urothelial carcinoma of the urinary system, etc. (5). Clinical imaging and pathological diagnosis after surgical resection can help to identify the primary lesion (40). In addition, it is necessary to diagnose ovarian primary squamous cell carcinoma and endometrial adenocarcinoma with scale changes, as they lack hair and sebum components (41). Immunohistochemistry may also be helpful for diagnosis and differential diagnosis.27

To identify the molecular biological characteristics and genomic abnormalities of MT-MCT, deoxyribonucleic acid (DNA) and microribonucleic acid (miRNA) analyses were performed (42). Cooke et al. reported that a total of 244 abnormalities were identified in 79 genes in SCC-MCT. TP53 was the most frequently altered gene in SCC (80%), followed by PIK3CA (52%) and CDKN2A (44%). The gene mutation in TP53 was associated with improved overall survival (43). The overall mutational burden of SCC-MCT is high, but MCT has a low mutation burden. SCC-MCTs share similar mutation profiles to SCC (43). Yoshida et al. analyzed comprehensive miRNA sequencing in SCC-MCTs and normal ovarian and mature teratoma tissues. Two miRNAs (miR-151a-3p and miR-378a-3p) were markedly upregulated, and two miRNAs (miR-26a-5p and miR-99a-5p) were markedly downregulated in cancer tissues. In addition, these findings were validated in fresh cancer tissues of patient-derived xenograft (PDX) models (44). Gene analysis research on MT-MCTs can help us better understand the cause of malignant transformation from teratomas to provide innovative thinking related to treatment and drug research.

As MT-MCTs are very rare, there is no standard treatment at present. The basic principle is that surgical treatment, early detection and complete resection can improve the survival rate (25, 45). For perimenopausal or postmenopausal women, total abdominal hysterectomy with bilateral salpingo-oophorectomy, omentectomy, pelvic lymphadenectomy are recommended (46). For early-stage women who have fertility requirements, the uterus and normal ovary could be preserved (25). Adjuvant chemotherapy mainly depends on the malignant pathological type of MCT. TC and TP are the most commonly used chemotherapy regimens (14). It has been reported that the 5-year survival rate of MT-MCTs with FIGO stage I is 95%, which is much better than that of patients with advanced-stage disease (47). Ruey-jien et al. reported that the 5-year survival rates of stage I, II, III and IV patients were 75.5%, 33.8%, 20.6% and 0%, respectively, after a follow-up of 188 patients (45). In our study, the 5-year OS rates in patients with FIGO stages I, II, III, and IV were 100.0%, 88.9%, 60%, and 0%, respectively. Therefore, tumor stage and optimal debulking are critical to survival outcomes (45).

## Conclusion

In conclusion, this retrospective study showed that the rate of malignant transformation in all ovarian MCTs was 0.30%. MT-MCTs mainly occur in elderly females, are rare and have a poor prognosis. Advanced FIGO stage is a risk factor for survival. Preoperative examination included positive tumor markers (CA125, CA199, and SCCAg), ultrasonic examination, pelvic enhanced MRI and CT. Although there is no standard treatment, cytoreductive debulking surgery and adjuvant chemotherapy could be considered. Perimenopausal and menopausal women with MCT should receive surgical treatment.

## Data Availability Statement

The raw data supporting the conclusions of this article will be made available by the authors, without undue reservation.

## Ethics Statement

All patients provided written informed consent under the approval of the Ethics Committee of PUMCH. The patients/participants provided their written informed consent to participate in this study. Written informed consent was obtained from the individual(s) for the publication of any potentially identifiable images or data included in this article.

## Author Contributions

Study conceptualization: L-yP, YJ; Study design: YL, MQ, YJ; Data acquisition: YL, MQ, YG, WW, LM; Quality control of data and algorithms: JY, J-yC, YS; Data analysis and interpretation: YL, MQ, Y-xW, H-wW, X-dL; Statistical analysis: YL, MQ; Manuscript preparation: YL, MQ; Manuscript editing: all authors; Manuscript review: YJ. All authors contributed to the article and approved the submitted version.

## Funding

This project was supported by the Non-profit Central Research Institute Fund of Chinese Academy of Medical Sciences (2021-PT320-001). Furthermore, this project was also supported by CAMS Innovation Fund for Medical Sciences (CIFMS-2017-I2M-1-002).

## Conflict of Interest

The authors declare that the research was conducted in the absence of any commercial or financial relationships that could be construed as a potential conflict of interest.

## Publisher’s Note

All claims expressed in this article are solely those of the authors and do not necessarily represent those of their affiliated organizations, or those of the publisher, the editors and the reviewers. Any product that may be evaluated in this article, or claim that may be made by its manufacturer, is not guaranteed or endorsed by the publisher.
