# Supplementary material for: 30-Year Experience With 22 Cases of Malignant Transformation Arising From Ovarian Mature Cystic Teratoma: A Rare Disease
Source: Front Oncol. 2022 May 9;12:842703. doi: 10.3389/fonc.2022.842703 (PMC9124836; doi:10.3389/fonc.2022.842703)
Supplement: Supplementary file 1 [file DataSheet_1.docx]

**Figure S1. The recurrence-free survival (A) and overall survival (B) of included patients with MT-MCT characterized by different MT histology.**
